# Quantification of the enzyme activities of iduronate-2-sulfatase, *N*-acetylgalactosamine-6-sulfatase and *N*-acetylgalactosamine-4-sulfatase using liquid chromatography-tandem mass spectrometry

**DOI:** 10.1016/j.ymgmr.2017.12.001

**Published:** 2017-12-21

**Authors:** Ryuichi Mashima, Mari Ohira, Torayuki Okuyama, Akiya Tatsumi

**Affiliations:** Department of Clinical Laboratory Medicine, National Center for Child Health and Development, 2-10-1 Okura, Setagaya-ku, Tokyo 157-8535, Japan

## Abstract

Mucopolysaccharidosis (MPS) is a genetic disorder characterized by the accumulation of glycosaminoglycans in the body. Of the multiple MPS disease subtypes, several are caused by defects in sulfatases. Specifically, a defect in iduronate-2-sulfatase (ID2S) leads to MPS II, whereas *N*-acetylgalactosamine-6-sulfatase (GALN) and *N*-acetylgalactosamine-4-sulfatase (ARSB) defects relate to MPS IVA and MPS VI, respectively. A previous study reported a combined assay for these three disorders in a 96-well plate using a liquid chromatography-tandem mass spectrometry (LC-MS/MS)-based technique (Kumar et al., Clin Chem 2015 61(11):1363-1371). In our study, we applied this methodology to a Japanese population to examine the assay precision and the separation of populations between disease-affected individuals and controls for these three disorders. Within our assay conditions, the coefficient of variation (CV, %) values for an interday assay of ID2S, GALN, and ARSB were 9%, 18%, and 9%, respectively (*n* = 7). The average enzyme activities of ID2S, GALN, and ARSB in random neonates were 19.6 ± 5.8, 1.7 ± 0.7, and 13.4 ± 5.2 μmol/h/L (mean ± SD, *n* = 240), respectively. In contrast, the average enzyme activities of ID2S, GALN, and ARSB in disease-affected individuals were 0.5 ± 0.2 (*n* = 6), 0.3 ± 0.1 (*n* = 3), and 0.3 (*n* = 1) μmol/h/L, respectively. The representative analytical range values corresponding to ID2S, GALN, and ARSB were 39, 17, and 168, respectively. These results raise the possibility that the population of disease-affected individuals could be separated from that of healthy individuals using the LC-MS/MS-based technique.

## 1 Introduction

Lysosomal storage disorders are characterized by defective enzyme activities in the lysosomes which lead to the accumulation of unprocessed biological components, such as mucopolysaccharides, oligosaccharides, sphingolipids, glycolipids, and oxidation products of cholesterol [1], [2]. Mucopolysaccharidosis (MPS) is a lysosomal storage disorder associated with the accumulation of glycosaminoglycans (GAGs) in the body [3]. Among the known MPS disease subtypes, MPS I and MPS II exhibit the accumulation of dermatan sulfate and heparan sulfate simultaneously, whereas MPS III, MPS IV, and MPS VI show an elevation in heparan sulfate, keratan sulfate, and dermatan sulfate levels, respectively, in each disease subtype-specific manner. Defective enzymes may be generated by nonsense, frame-shift, missense, and other genetic mutations, which can lead to pathogenic manifestations [4]. To treat these disorders, a recombinant human enzyme can be infused into the affected individuals; this procedure is called enzyme replacement therapy [5]. Accumulating evidence suggests that it is beneficial to identify affected individuals during newborn screening due to more effective therapeutic outcomes [6]. For example, in the case of infantile-onset Pompe disease, treatment outcomes of affected individuals improved after the newborn screening program was implemented [7]. Similarly, newborn screening for MPS I, a prototypical MPS disease subtype, has been implemented in Taiwan, and some pilot studies have been conducted in other countries [8], [9], [10].

Two types of assays are used to diagnose MPS. The first type involves the measurement of enzyme activities through the accumulation of an enzyme-specific reaction product using artificial substrates [11]. While fluorescence-active substrates have been used for this type of assay in the past decade, several recent studies have highlighted the advantage of using a newly developed substrate which can be readily quantified using tandem mass spectrometry (MS/MS)-based techniques [10], [12], [13]. Most importantly, these new substrates allow the measurement of the activities of multiple enzymes simultaneously via multiple reaction monitoring, leading to an increasing sample throughput in the laboratory. In addition, this new assay detects a disease-affected individual with low enzyme activity from a large number of normal controls. An analytical range is a measure defined by the ratio of enzyme reaction products in control samples to those in the blank sample by Kumar et al. [14]. Within examined studies the liquid chromatography (LC)-MS/MS-based assay provides a larger analytical range compared to a fluorometric assay [10], [14], [15]. The subsequently reported data of analytical ranges using LC-MS/MS appeared to be similar to those of several other studies [12], [16].

The pathogenesis of MPS II, IVA, and VI is induced by the defective enzyme activity of iduronate-2-sulfatase (ID2S), *N*-acetylgalactosamine-6-sulfatase (GALN), and *N*-acetylgalactosamine-4-sulfatase (ARSB), respectively. The prototypical combination of substrate and internal standard for these disorders was first synthesized in 2007–2011 [17], [18], [19], [20], [21]. Due to the smaller ion counts of the original reagents, an improved set of reagents that generates much larger ion counts has been further synthesized [14], [22]. Using these new reagents, Kumar et al. reported that the analytical range for ID2S, GALN, and ARSB using LC-MS/MS has been improved, compared to that using fluorometry [14]. In a more recent study, these new reagents were used in a combination with other enzyme for lysosomal strorage disorders (LSDs) [12]. The authors understand that these two North American studies are the only ones that used these new reagents [12], [14]. Therefore, we have applied this analytical technique for a Japanese population to examine the separation of populations between controls and disease-affected individuals for MPS-II, IVA, and VI.

## 2 Experimental

### 2.1 Materials

The reagents required for MPS enzyme assay (ID2S, GALN, and ARSB) and reaction buffer were obtained from PerkinElmer (Waltham, MA, USA). Acetonitrile and methanol were purchased from Thermo Fisher Scientific (Tokyo, Japan). Deionized water from a Milli-Q water system was obtained from Millipore (Milford, MA, USA). Formic acid was purchased from Kanto Chemical (Tokyo, Japan). A set of dried blood spot (DBS) for quality control (QC) with high, middle, low, or base activity was obtained from PerkinElmer. All of the other reagents used in this study were of the highest commercial grade available.

### 2.2 Approval by the institutional research ethics board

This study was approved by the Research Ethics Board of the National Center for Child Health and Development.

### 2.3 DBS

DBS was stored at − 20 °C as reported previously [23]. Neonatal specimens were used as the healthy control. None of the affected individuals was a neonate, and all received enzyme replacement therapy.

### 2.4 MPS II/IVA/VI assay

This assay was performed in accordance with previous studies, but with slight modifications [12], [14]. Briefly, the assay cocktail contained known concentrations of the substrate: ID2S to detect MPS II (0.5 mM), GALN to detect MPS IVA (1 mM), and ARSB to detect MPS VI (1 mM). Further, it also contained internal standards for ID2S (5 μM), GALN (5 μM), and ARSB (5 μM). Enzyme reactions were performed in 50 mM of ammonium acetate (pH 5.0), containing 7.5 mM of barium acetate, 5 mM of cerium acetate, and 2 mM of *O*-(2-Acetamido-2-deoxy-d-glucopyranosylidene)amino *N*-phenyl carbamate as an inhibitor for *N*-acetylglucosaminidase. All the assays were carried out with a 3-mm punch using 30 μL of the assay cocktail in a polypropylene 96-well plate (260,252; Thermo Fisher Scientific, Tokyo, Japan); the 96-well plate containing the cocktail was incubated at 37 °C for 20 h. To terminate the reaction, a mixture of methanol/ethyl acetate (50/50, 0.1 mL) was added to the wells. Subsequently, to extract the enzyme reaction products, ethyl acetate (0.4 mL) and 0.5 M sodium chloride (0.2 mL) were added and mixed 20 times using a pipette. After centrifuging these plates at 700 ×* g* for 5 min at room temperature (PlateSpin II; Kubota, Tokyo, Japan), an aliquot of the organic layer (0.2 mL) was collected and injected into a fresh 96-well plate followed by evaporation using a nitrogen stream. Finally, the reaction products were reconstituted using a solution of acetonitrile/water/formic acid (80/20/0.1, 0.15 mL). In this study, we estimated that a 3-mm DBS punch contained 3.1 μL of whole blood; enzyme activity was calculated in μmol/h/L blood, as reported previously [14].

### 2.5 LC-MS/MS

An aliquot (1 μL) was injected into a tandem mass spectrometer (Quattro Premier XE; Waters, Milford, MA, USA) equipped with an ACQUITY UPLC and an autosampler (Waters, Milford, MA, USA). Aliquots of the samples were injected into an analytical column (ACQUITY CSH C18; 2.1 mm inner diameter, 30 mm length, 1.7 μm particle size) equilibrated with 60% mobile phase A (0.2% formic acid in 5% acetnitrile/95% water) and 40% mobile phase B (0.2% formic acid in acetonitrile) at a flow rate of 0.6 mL/min at 40 °C. The enzyme reaction products and internal standards were eluted with 40% B for 0–0.1 min, 40%–80% B for 0.1–1.0 min, 80% B for 1.0–1.25 min, and 40% B for 1.26–2.0 min. The results were acquired using MassLynx V4.1 software (Waters, Milford, MA, USA). Other analytical conditions employed in the study are described in Supplementary Tables 1–6.

## 3 Results

For both screening and diagnosis, it is essential to quantify the accumulation of enzyme reaction products for each disease-causing enzyme. This is particularly important in MPS because of multiple disease subtypes. In fact, accumulation of the enzyme reaction products of ID2S for MPS II, GALN for MPS IVA, and ARSB for MPS VI were readily detected in reaction mixtures with a DBS for QC with high enzyme activity (QC High) and one from a random neonate even before the optimization of analytical settings (Supplementary Figs. 1–3). Based on this preliminary observation, we further examined the MS/MS conditions for enzyme reactions in terms of the flow rate of N_2_ gas and the temperature of the desolvation gas (Supplementary Fig. 4). For high throughput screening, several LC conditions were additionally tested using a commercially available analytical column charged surface hybrid (CSH C18; Waters, Milford, MA, USA) (1.7 μm, 2.1 × 30 mm) for UPLC (Supplementary Table 7). Under these established conditions, we found that the accumulation of enzyme reaction products corresponding to ID2S, GALN, and ARSB was linear in the whole blood concentration range of 0%–100% within our assay precision limits (R^2^ > 0.99, Supplementary Fig. 5). We also found that the CV values for an interday assay (*n* = 7) of ID2S, GALN, and ARSB were 9%, 18%, and 9%, respectively (Table 1). Similarly, the values of coefficient of variation (CV) for an intraday assay (*n* = 5) of ID2S, GALN, and ARSB were 12%, 4%, and 5%, respectively.

**Table 1:** Interday and intraday CV values for ID2S, GALN, and ARSB assay using LC-MS/MS.

	ID2S	GALN	ARSB
Interday parameter			
Mean (μmol/h/L)	12.1	3.2	22.7
SD (μmol/h/L)	1.1	0.6	2.0
CV (%)	9	18	9
*n*	7	7	7
Intraday parameter			
Mean (μmol/h/L)	12.2	4.1	24.0
SD (μmol/h/L)	1.4	0.2	1.20
CV (%)	12	4	5
*n*	5	5	5

Enzyme activities were determined using a QC DBS with high enzyme activity provided by PerkinElmer.

**Fig. 1:**
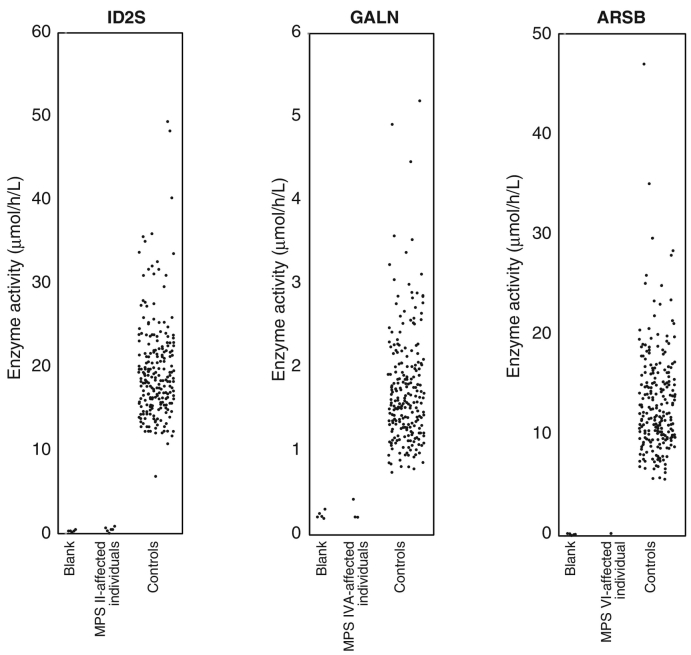
Enzyme activities in random neonates and disease-affected individuals measured using LC-MS/MS. The enzyme activities of DBSs in blank (*n* = 5), random neonates (*n* = 240), MPS II-affected individuals (*n* = 6), MPS IVA-affected individuals (*n* = 3), and an MPS VI-affected individual (*n* = 1) were examined.

As shown in Fig. 1, the elevation of calculated enzyme activities corresponding to ID2S, GALN, and ARSB were apparently higher in the population of random neonates when compared to the activities in filter paper blank samples. Specifically, the enzyme activities (mean ± SD) of ID2S in blank samples, random neonates, and MPS II-affected individuals were 0.8 ± 1.1 (median 0.0; max 2.42; min 0.0; *n* = 5), 19.6 ± 5.8 (median 18.45; max 49.34; min 6.89; *n* = 240), and 0.5 ± 0.2 (median 0.42; max 0.84; min 0.16; *n* = 6), respectively (Fig. 1 *Left*). Similarly, the enzyme activities (mean ± SD) of GALN in blank samples, random neonates, and MPS IVA-affected individuals were 0.2 ± 0.0 (median 0.23; max 0.31; min 0.21; *n* = 5), 1.7 ± 0.7 (median 1.58; max 5.20; min 0.75; *n* = 240), and 0.3 ± 0.1 (median 0.22; max 0.42; min 0.22; *n* = 3), respectively (Fig. 1 *Middle*). Finally, the enzyme activities (mean ± SD) of ARSB in blank samples, random neonates, and a disease-affected individual were 0.1 ± 0.1 (median 0.16; max 0.21; min 0.07; *n* = 5), 13.4 ± 5.2 (median 12.25; max 46.93; min 5.69; *n* = 240), 0.3 (*n* = 1), respectively (Fig. 1 *Right*). In all three diseases, the median values were lower than the means, indicating that the enzyme activity of the tested population showed a bell-shaped distribution with an extended tail at the higher range (Supplementary Fig. 6).

To exclude the possibility of whether a DBS with low enzyme activity may be misdiagnosed as disease-affected, we provided all three enzyme activities of disease-affected individuals together with those of a set of QC DBS as a control for properly stored standard materials. First, we calculated the enzyme activity for ID2S, GALN, and ARSB in DBS of QC High, which contains a normal level of leukocyte (100%) (Table 2 *Left bottom*). Similarly, in the DBS of QC Middle with 50% leukocyte, there were 6.4 μmol/h/L of ID2S, 1.9 μmol/h/L of GALN, and 11.3 μmol/h/L of ARSB enzyme activity, indicating that the calculated enzyme activities for the three enzymes in DBS of QC Middle were 47%–53%. Similarly, a DBS of QC Low showed 0.9 μmol/h/L of ID2S, 0.4 μmol/h/L of GALN, and 2.2 μmol/h/L of ARSB enzyme activity, indicating that this DBS contained 7%–9% of enzyme activities compared to those of QC High. Importantly, although the levels of enzyme activity depend on leukocyte content, the relative enzyme activities of each enzyme in a DBS are expected to be the same. For example, the relative enzyme activities for ID2S to GALN, GALN to ARSB, and ARSB to ID2S in a DBS of QC High were calculated as 3.01, 17, and 197, respectively (Table 2 *Center bottom*). These values remain unaltered regardless of leukocyte content, leading to the fact that the average relative enzyme activities for ID2S to GALN, GALN to ARSB, and ARSB to ID2S were calculated as 2.86, 17, and 212, respectively. For further comparison, these values were expressed as 100% of QC_average_ (Table 2 *Center bottom*).

**Table 2:** Enzyme activities of ID2S, GALN, and ARSB in disease-affected individuals.

Sample	ID	Enzyme activity			Relative enzyme activity					
		ID2S	GALN	ARSB	ID2S/GALN	GALN/ARSB	ARSB/ID2S	ID2S/GALN	GALN/ARSB	ARSB/ID2S
		(μmol/h/L)				(Ratio)			(%QC_average_)	
MPS II	1	0.7	1.8	8.7	0.40	0.21	12	14	1.2	6
	2	0.2	1.1	3.7	0.15	0.30	23	5	1.7	11
	3	0.4	2.0	6.7	0.20	0.30	17	7	1.8	8
	4	0.8	1.4	4.5	0.62	0.30	5	22	1.8	3
	5	0.4	1.8	7.1	0.23	0.26	17	8	1.5	8
	6	0.4	1.3	10.0	0.32	0.13	24	11	0.8	11
MPS IVA	1	37.5	0.4	4.5	89	0.09	0.12	3118	0.55	0.06
	2	17.3	0.2	7.6	79	0.03	0.44	2752	0.17	0.21
	3	17.6	0.2	5.5	80	0.04	0.31	2795	0.24	0.15
MPS VI	1	24.9	2.4	0.3	10	9	0.01	357	51	0.005
QC High		12.2	4.1	24.0	3.01	17	197	105	99	93
QC Middle		6.4	1.9	11.3	3.32	17	177	116	100	84
QC Low		0.9	0.4	2.2	2.26	17	260	79	100	123
QC_average_		NA	NA	NA	2.86	17	212	100	100	100

Each QC High, Middle, and Low DBS contains 100, 50, and 5% of leukocytes with the same hematocrit values to normal blood.

All disease-affected individuals received enzyme replacement therapy at the time of sample collection.

Based on these values, each relative enzyme activity for three enzymes in all disease-affected individuals can be standardized. As shown, six MPS II-affected individuals exhibited 0.2–0.8 μmol/h/L of ID2S enzyme activity corresponding to 0.15–0.62 of relative enzyme activity of ID2S to GALN (Table 2 *Center*). When we normalized these values to the relative enzyme activity of ID2S to GALN (i.e., 2.86 in this case), we found the relative enzyme activities of ID2S for six MPS II-affected individuals were within 5%–22% (Table 2 *Right*). Given that %QC_average_ is defined as 100%, these disease-affected values were low. Similarly, the GALN enzyme activity in MPS IVA-affected individuals was 0.2–0.4 μmol/h/L, corresponding to 0.03–0.09 of relative enzyme activity of GALN to ARSB (0.17%–0.55% compared to QC_average_). ARSB activity for an MPS VI-affected individual in this study was 0.3 μmol/h/L, indicating that the ratio of ARSB to ID2S was 0.01 (0.005% of QC_average_ value). Apparently, the relative enzyme activities for GALN in the DBS of MPS IVA-affected individuals and for ARSB in that of MPS VI-affected individuals were lower than that of the other enzymes. Overall, these results clearly demonstrated that the relative enzyme activity in DBS from disease-affected individuals was specifically attenuated in a disease-dependent manner.

The analytical range is a measure defined by the ratio of enzyme reaction products in control samples to the reaction products in the blank sample. We calculated the analytical ranges based on the enzyme activities in QC DBS with high enzyme activity. The analytical ranges for ID2S, GALN, and ARSB were 39, 17, and 168, respectively (Table 3).

**Table 3:** Analytical ranges for ID2S, GALN, and ARSB assay using LC-MS/MS method.

Method	LC-MS/MS			Fluorimetry
Investigator	Mashima R et al	Kumar AB et al.	Liu Y et al.	Kumar AB et al.
	(Ranges)			
ID2S	39	430–577	243	8.7–11.4
GALN	17	85–119	909	6.5–9.14
ARSB	168	143–188	102	5.94–7.8
Reference	This study	[14]	[12]	[14]

## 4 Discussion

The use of the measurement of MPS enzyme activity to identify disease-causing enzymes is more advantageous for both screening and diagnostic purposes when compared to the measurement of GAGs as biomarkers (Reviewed in [11]). For these purposes, it is important that the population of disease-affected individuals be separated from that of controls. This can be achieved by the assay with reasonable accuracy at low enzyme activity. A potential failure of this is associated with improper separation of these two populations, leading to additional biochemical and/or genetic testing. An MS/MS-based technique allows much wider analytical ranges compared to fluorescence-based assays, making this assay so much more attractive for screening and diagnostics [10], [14], [15], [16]. We used neonatal DBS for controls, whereas no disease-affected individuals were neonate. This is an important issue, because, in general, the average enzyme activity for LSD may vary depending on enzyme and age. For example, there is a recent study demonstrating that adult DBSs show lower enzyme activity for ARSB, whereas they show higher enzyme activity for GALN [12]. Apart from these two examples, enzyme activities for ID2S in neonates and adults exhibited no apparent difference. Given that adults show lower enzyme activity than neonates, such as for ARSB, the comparison of the populations between healthy neonate controls and disease-affected adults shows a much clearer separation than the other two proper comparisons. In this study, essentially, we were able to demonstrate the almost similar enzyme activities for ID2S, GALN, and ARSB as previously reported by Kumar et al. [14]. Consistent with the previous study, GALN showed the lowest enzyme activity among these three enzymes. As shown in Fig. 1, we were able to demonstrate the separation of populations between disease-affected individuals and neonates; thus, further study will be required to establish more validated cut-off values using this methodology.

Relative enzyme activity in DBS is a good measure to assess whether the DBS of interest derives from a disease-affected individual or has all low enzyme activities. In fact, an earlier study demonstrated that several enzyme activities of lysosomal storage disorders in DBS might decrease when the DBS remains wet [23]. Thus, there is a substantial demand to detect such DBSs. Collaborative Laboratory Integrated Reports software accommodates multiple MS/MS data to identify DBS with abnormal enzyme activity [24]. Our data showed that identification of such DBS seemed also possible through calculated relative enzyme activities by multiplex data (Table 2). Relative enzyme activity in a disease-affected individual shows selectively and abnormally low values in a disease-dependent manner, whereas DBS with low enzyme activity in a non-selective manner shows higher relative enzyme activities compared to that from disease-affected individuals.

Essentially, the separation of the populations with controls and disease-affected individuals is a key element of both screening and diagnosis. The analytical range is a measure defined by the ratio of enzyme reaction products in control samples to the reaction products in the blank sample [14]. A wider analytical range value enables quantification of lower enzyme activity, leading to the separation of a population with disease-affected individuals from that with controls. At this stage, three studies reported that the analytical range using an MS/MS assay is wider than that using a fluorometric assay [10], [14], [15]. Recently, several studies have suggested that it is important that the population of disease-affected individuals would be better separated from that of individuals with pseudodeficient alleles. For example, it is well-known that the Asian population has approximately 3% of pseudodeficiency alleles in the *GAA* gene corresponding to G576S mutation [26]. In fact, a recent study demonstrated that the LC-MS/MS-based technique allows the separation of populations between controls and individuals with peudodeficiency alleles in the screening of α-glucosidase activity [27]. A similar conclusion was reported in a different study in a Japanese population with a limited number of specimens [16]. Thus, a larger analytical range offered by the MS/MS-based technique appears to be strongly linked to the potential for the separation of populations between disease-affected individuals and controls.

The diagnosis of MPS with specific biomarkers may require an elevation in the GAG levels followed by selective accumulation of at least one of the following compounds— dermatan sulfate, heparan sulfate, or keratan sulfate—in affected individuals [28], [29], [30], [31], [32]. Genetic testing of suspected individuals will be performed subsequently only when both of them are positive. To prove the selective accumulation of disease-specific disaccharides, GAGs in the biological samples need to be digested by chemical cleavage [28], [29], [30], [31], [32]. Alternatively, this process can be performed using digestion enzymes specific for dermatan sulfate, heparan sulfate, and keratan sulfate [33], [34]. It is well-known that MPS III, IV, and VI lead to the elevation of heparan sulfate, keratan sulfate, and dermatan sulfate levels, respectively. In contrast, MPS I and MPS II lead to simultaneous elevations in dermatan sulfate and heparan sulfate levels [28], [31]. In these two disorders, the enzyme activities of α-L-iduronidase and ID2S must be quantified for diagnosis with MPS I and MPS II, respectively.

In conclusion, we applied the recently published LC-MS/MS-based technique to test enzyme activity of ID2S for MPS II, GALN for MPS IVA, and ARSB for MPS VI in a Japanese population. When multiplex assays for lysosomal storage disorders with different combinations of enzymes are to be developed, the optimal pH for each enzyme under the assay conditions may vary. In such cases, careful examination may be required. Relative enzyme activity can be used for the identification of disease-affected individuals, especially if all enzyme activities in DBS show lower values for various reasons. A proper experimental design selecting age-matched individuals is preferred, while the comparison of the populations between controls and disease-affected individuals offers a clinical rationale for the estimation of cut-off values in both screening and diagnosis at this stage.

## Appendix A Supplementary data

Supplementary material Image 1

Supplementary figures Image 2
