# Supplementary material for: Quantification of the enzyme activities of iduronate-2-sulfatase, N-acetylgalactosamine-6-sulfatase and N-acetylgalactosamine-4-sulfatase using liquid chromatography-tandem mass spectrometry
Source: Mol Genet Metab Rep. 2017 Dec 21;14:36–40. doi: 10.1016/j.ymgmr.2017.12.001 (PMC5758840; doi:10.1016/j.ymgmr.2017.12.001)
Supplement: Supplementary file 1 — Supplementary material [file mmc1.docx]

**Supplementary Figure legends**

**Supplementary Fig. 1**

*Upper,* the formation of enzyme reaction products in QC high (left), an MPS II-affected individual (center), and a random neonate (right). *Lower,* the amount of internal standard in QC high (left), an MPS II-affected individual (center), and a healthy individual (right). An arrowhead indicates the peak of ID2S-derived enzyme reaction product. A star indicated the peak of substrate for ID2S enzyme assay appeared due to thermal decomposition.

**Supplementary Fig. 2**

*Upper,* the formation of enzyme reaction products in QC high (left), an MPS IVA-affected individual (center), and a random neonate (right). *Lower,* the amount of internal standard in QC high (left), an MPS IVA-affected individual (center), and a healthy individual (right). An arrowhead indicates the peak of GALN-derived enzyme reaction product. A star indicated the peak of substrate for GALN enzyme assay appeared due to thermal decomposition.

**Supplementary Fig. 3**

*Upper,* the formation of enzyme reaction products in QC high (left), an MPS VI-affected individual (center), and a random neonate (right). *Lower,* the amount of internal standard in QC high (left), an MPS VI-affected individual (center), and a healthy individual (right). An arrowhead indicates the peak of ARSB-derived enzyme reaction product. A star indicated the peak of substrate for ARSB enzyme assay appeared due to thermal decomposition.

**Supplementary Fig. 4**

The peak area of internal standards for ID2S, GALN, and ARSB under different conditions in MS/MS measurement. *Upper,* the peak areas for internal standards at the flow rate of 300 L/h at 150, 250, 350, 450, and 500 °C of desolvation temperature was shown. *Middle,* the peak areas for internal standards at the flow rate of 500 L/h at 150, 250, 350, 450, and 500 °C of desolvation temperature was shown. *Lower,* the peak areas for internal standards at the flow rate of 800 L/h at 150, 250, 350, 450, and 500°C of desolvation temperature was shown. Note that N_2_ gas was used in all examination.

**Supplementary Fig. 5**

Calibration curves for MPS enzymes in LC-MS/MS-based assay. *Upper,* accumulation of ID2S enzyme activity in QC High/Middle/Low (100%, 50%, and 10% of enzyme activity). *Middle,* accumulation of GALN enzyme activity in QC High/Middle/Low (100%, 50%, and 10% of enzyme activity). *Lower,* accumulation of ARSB enzyme activity in QC High/Middle/Low (100%, 50%, and 10% of enzyme activity). Enzyme activity was expressed in μmol/h/L in blood.

**Supplementary Fig. 6**

Frequency distribution of enzyme activity determined using LC-MS/MS-based technique (*n* = 240). *Upper,* distribution of ID2S enzyme activity in random neonates. *Middle,* distribution of GALN enzyme activity in random neonates. *Lower,* distribution of ARSB enzyme activity in random neonates.

**Supplementary Table 1**

UPLC method for the quantification of P/IS for ID2S, GALN, and ARSB in the DBS using LC-MS/MS.

| LC | ACQUITY (Waters) |
| --- | --- |
| MS | Quattro Premier XE (Waters) |
| Column | ACQUITY CSH C18 (Waters) |
|  | Particle diameter:1.7 μm |
|  | Internal diameter: 2.1 mm |
|  | Length: 30 mm |
| Column temperature | 40°C |
| Weak wash solvent | Acetonitrile/water (20/80) |
| Strong wash solvent | Acetonitrile |
| Mobile phase A | 0.2 % formic acid in 5 % acetonitrile/95 % water |
| Mobile phase B | 0.2 % formic acid in acetonitrile |
| Gradient (%B) | 0 - 0.1 min: 40 %B |
|  | 0.1 – 1.0 min: 40 % - 80 %B |
|  | 1.0 – 1.25 min: 80 %B |
|  | 1.26 – 2.0 min: 40 %B |
| Flow rate | 0.6 mL/min |
| Injection volume | 1.0 μL |
| Sample loop volume | 10 μL |
| Autosampler temperature | 4 °C |

**Supplementary Table 2**

Instrument parameters for ID2S, GALN, and ARSB assays using a Waters Quattro Premier XE UPLC-MS/MS system.

| Interface | ESI |
| --- | --- |
| Polarity | Positive |
| Capillary voltage | 2.0 kV |
| Source temperature | 120 °C |
| Desolvation temperature | 500 °C |
| Flow rate of cone gas | 50 L/hr |
| Flow rate of desolvation gas | 500 L/hr |
| Analyzing mode | MRM |
| Dwell time | 20 ms |
| Data format | Centroid |

MRM, multiple reaction monitoring.

**Supplementary Table 3**

MRM parameters for ID2S, GALN, and ARSB assay using a Waters Quattro Premier XE UPLC-MS/MS system.

|  | Precursor ion  (*m/z*) | Product ion  (*m/z*) | Cone  (V) | Collision energy  (V) |
| --- | --- | --- | --- | --- |
| ID2S-IS | 649.30 | 364.30 | 25 | 20 |
| ID2S-P | 644.25 | 359.30 | 25 | 20 |
| ID2S-S | 724.30 | 359.23 | 26 | 20 |
| GALN-IS | 690.40 | 378.30 | 35 | 25 |
| GALN-P | 685.40 | 373.30 | 25 | 20 |
| GALN-S | 765.30 | 373.30 | 15 | 20 |
| ARSB-IS | 662.40 | 350.30 | 25 | 25 |
| ARSB-P | 657.35 | 345.20 | 25 | 20 |
| ARSB-S | 737.30 | 345.20 | 15 | 23 |

IS, internal standard.

**Supplementary Table 4**

UHPLC method for the quantification of P/IS for ID2S, GALN, and ARSB in the DBS using LC-MS/MS.

| LC | Nexara (Shimadzu) |
| --- | --- |
| MS | LC8030plus (Shimadzu) |
| Column | MonoTower C18 (GL Sciences) |
|  | Internal diameter: 3 mm |
|  | Length: 100 mm |
| Column temperature | 40 °C |
| Wash solvent | Acetonitrile |
| Mobile phase A | 0.2 % formic acid in water |
| Mobile phase B | Acetonitrile |
| Elution | Isocratic 80 %B |
| Flow rate | 0.6 mL/min |
| Injection volume | 5 μL |
| Autosampler temperature | 4 °C |

**Supplementary Table 5**

Instrument parameters for ID2S, GALN, and ARSB assays using a Shimadzu LC8030plus.

| Interface | ESI |
| --- | --- |
| Polarity | Positive |
| Capillary voltage | 2.0 kV |
| Heatblock temperature | 400 °C |
| Desolvation line temperature | 250 °C |
| Flow rate of nebulizer gas | 3 L/min |
| Flow rate of drying gas | 15 L/min |
| Analyzing mode | MRM |
| Dwell time | 20 ms |

MRM, multiple reaction monitoring.

**Supplementary Table 6**

MRM parameters for ID2S, GALN, and ARSB assay using a Shimadzu LC8030plus.

|  | Precursor ion  (*m/z*) | Product ion  (*m/z*) | Q1 Pre Bias  (V) | CE  (V) | Q3 Pre Bias  (V) |
| --- | --- | --- | --- | --- | --- |
| ID2S-IS | 649.15 | 364.15 | -24 | -21 | -24 |
| ID2S-P | 644.15 | 359.15 | -24 | -21 | -24 |
| ID2S-S | 724.00 | 359.10 | -26 | -26 | -23 |
| GALN-IS | 690.00 | 378.15 | -28 | -27 | -24 |
| GALN-P | 685.00 | 373.15 | -28 | -27 | -24 |
| GALN-S | 765.05 | 373.10 | -28 | -29 | -25 |
| ARSB-IS | 662.00 | 350.10 | -28 | -23 | -23 |
| ARSB-P | 657.00 | 345.10 | -28 | -23 | -23 |
| ARSB-S | 737.10 | 345.05 | -26 | -29 | -22 |

CE, collision energy.

**Supplementary Table 7**

Retention times of S/IS for 3 LSD enzymes using gradient elution.

| Run | #1 | #2 | #3 | #4 | #5 | #6 | #7 | #8 | #9 | #10 | #11 | #12 |
| --- | --- | --- | --- | --- | --- | --- | --- | --- | --- | --- | --- | --- |
|  |  |  |  |  |  |  |  |  |  |  |  |  |
| Temp (°C) | 40 | 45 | 50 | 60 | 40 | 45 | 50 | 60 | 40 | 45 | 50 | 60 |
| Flow rate (mL/min) | 0.6 | 0.6 | 0.6 | 0.6 | 0.6 | 0.6 | 0.6 | 0.6 | 0.6 | 0.6 | 0.6 | 0.6 |
| Compound | Retention time | | | | | | | | | | |  |
|  | (min) | | | | | | | | | | |  |
| ID2S_IS | 0.26 | 0.25 | 0.26 | 0.24 | 0.25 | 0.26 | 0.26 | 0.25 | 0.66 | 0.66 | 0.65 | 0.64 |
| ID2S _S | 0.62 | 0.59 | 0.58 | 0.53 | 0.56 | 0.55 | 0.55 | 0.50 | 0.90 | 0.88 | 0.86 | 0.84 |
| GALN_IS | 0.26 | 0.25 | 0.28 | 0.26 | 0.26 | 0.26 | 0.28 | 0.27 | 0.67 | 0.66 | 0.68 | 0.67 |
| GALN_S | 0.64 | 0.62 | 0.63 | 0.59 | 0.59 | 0.57 | 0.57 | 0.54 | 0.91 | 0.90 | 0.88 | 0.88 |
| ARSB_IS | 0.20 | 0.20 | 0.20 | 0.21 | 0.20 | 0.20 | 0.20 | 0.20 | 0.56 | 0.55 | 0.55 | 0.55 |
| ARSB_S | 0.49 | 0.48 | 0.48 | 0.44 | 0.47 | 0.48 | 0.46 | 0.43 | 0.82 | 0.81 | 0.80 | 0.77 |
|  |  |  |  |  |  |  |  |  |  |  |  |  |
|  |  |  |  |  |  |  |  |  |  |  |  |  |
|  | Gradient program | | | | | | | | | | |  |
|  | Time (min): B (%) | | | | | | | | | | |  |
|  | 0.01: 40 | 0.01: 40 | 0.01: 40 | 0.01: 40 | 0.01: 40 | 0.01: 40 | 0.01: 40 | 0.01: 40 | 0.01: 20 | 0.01: 20 | 0.01: 20 | 0.01: 20 |
|  | 0.25: 40 | 0.25: 40 | 0.25: 40 | 0.25: 40 | 0.10: 40 | 0.10: 40 | 0.10: 40 | 0.10: 40 | 0.10: 20 | 0.10: 20 | 0.10: 20 | 0.10: 20 |
|  | 1.00: 80 | 1.00: 80 | 1.00: 80 | 1.00: 80 | 1.00: 80 | 1.00: 80 | 1.00: 80 | 1.00: 80 | 1.00: 80 | 1.00: 80 | 1.00: 80 | 1.00: 80 |
|  | 1.25: 80 | 1.25: 80 | 1.25: 80 | 1.25: 80 | 1.25: 80 | 1.25: 80 | 1.25: 80 | 1.25: 80 | 1.25: 80 | 1.25: 80 | 1.25: 80 | 1.25: 80 |
|  | 1.26: 40 | 1.26: 40 | 1.26: 40 | 1.26: 40 | 1.26: 40 | 1.26: 40 | 1.26: 40 | 1.26: 40 | 1.26: 20 | 1.26: 20 | 1.26: 20 | 1.26: 20 |
|  | 2.00: E | 2.00: E | 2.00: E | 2.00: E | 2.00: E | 2.00: E | 2.00: E | 2.00: E | 2.00: E | 2.00: E | 2.00: E | 2.00: E |

An ACQUITY CSH C18 column (2.1 × 30 mm, 1.7 μm, Waters) was used. E, end.
