# Supplementary figures and images for: Quantification of the enzyme activities of iduronate-2-sulfatase, N-acetylgalactosamine-6-sulfatase and N-acetylgalactosamine-4-sulfatase using liquid chromatography-tandem mass spectrometry
Source: Mol Genet Metab Rep. 2017 Dec 21;14:36–40. doi: 10.1016/j.ymgmr.2017.12.001 (PMC5758840; doi:10.1016/j.ymgmr.2017.12.001)

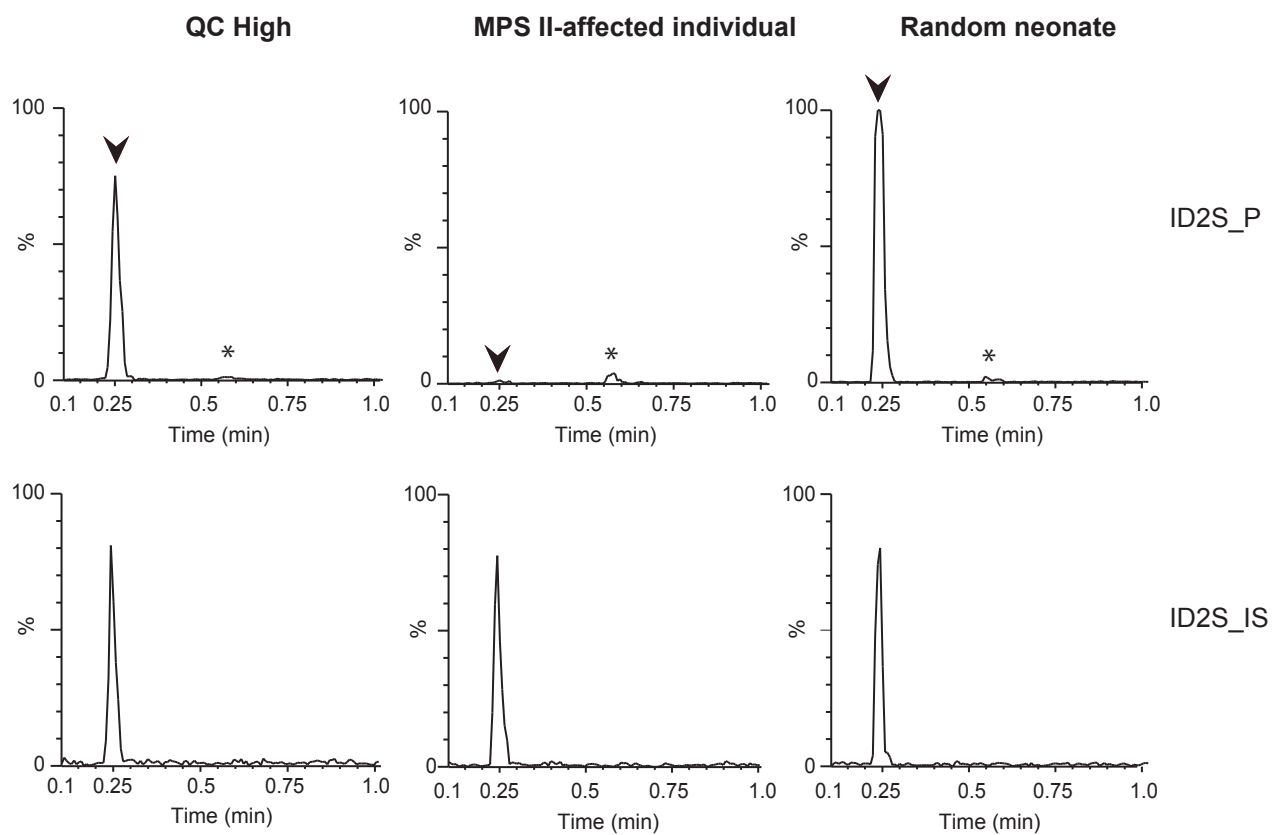

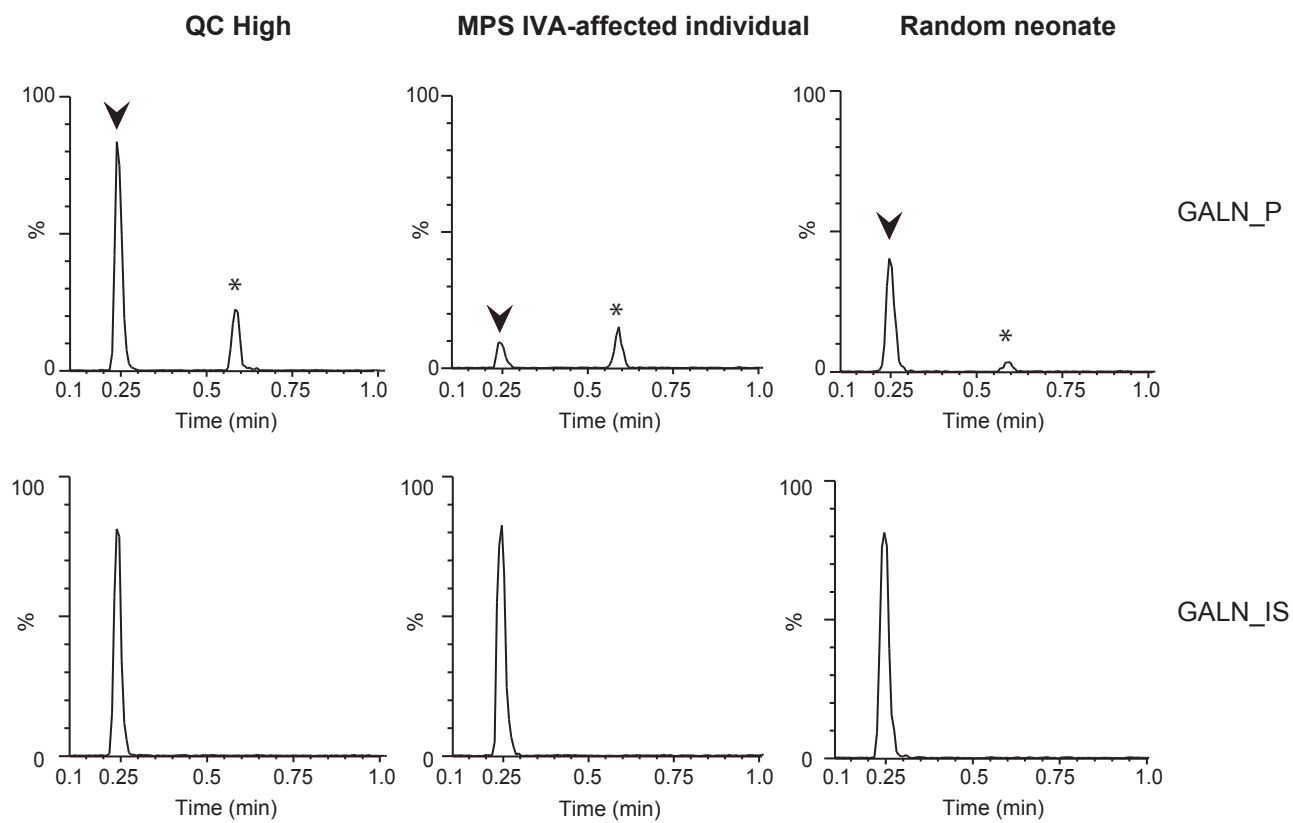

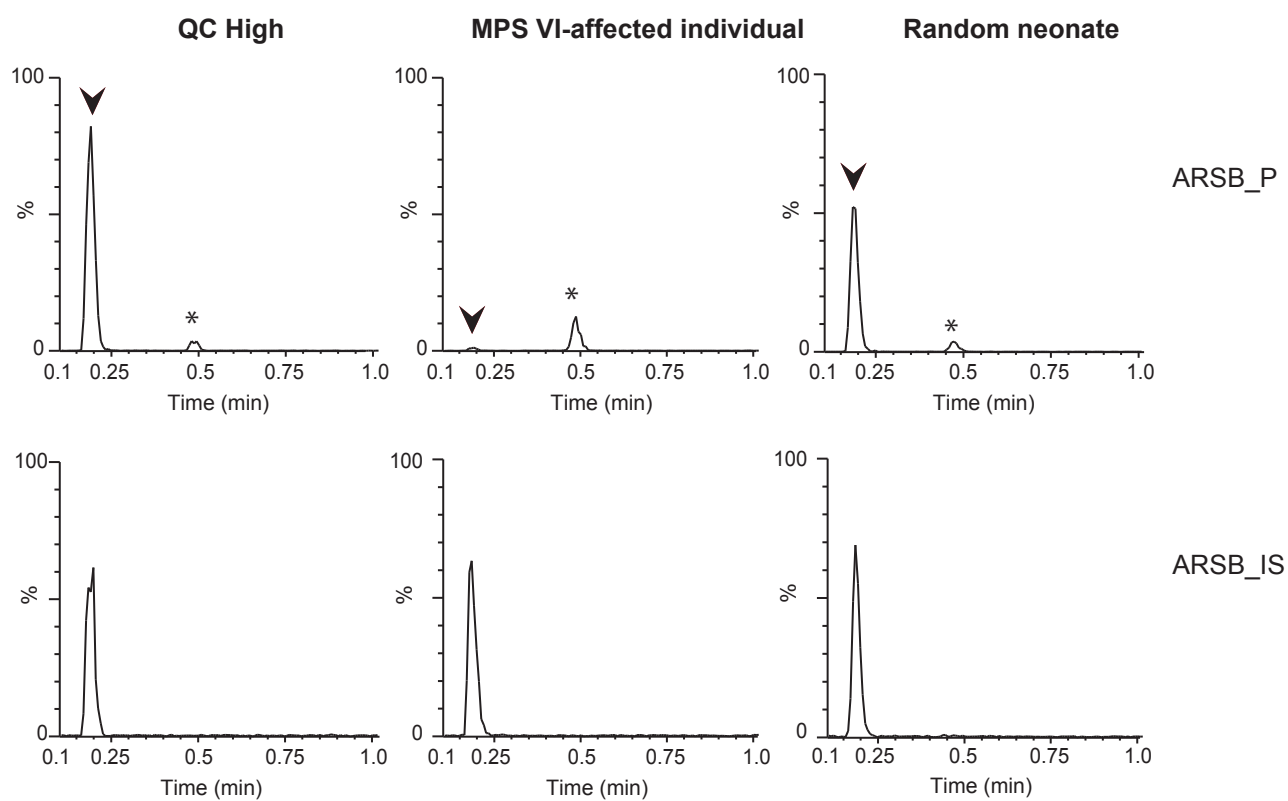

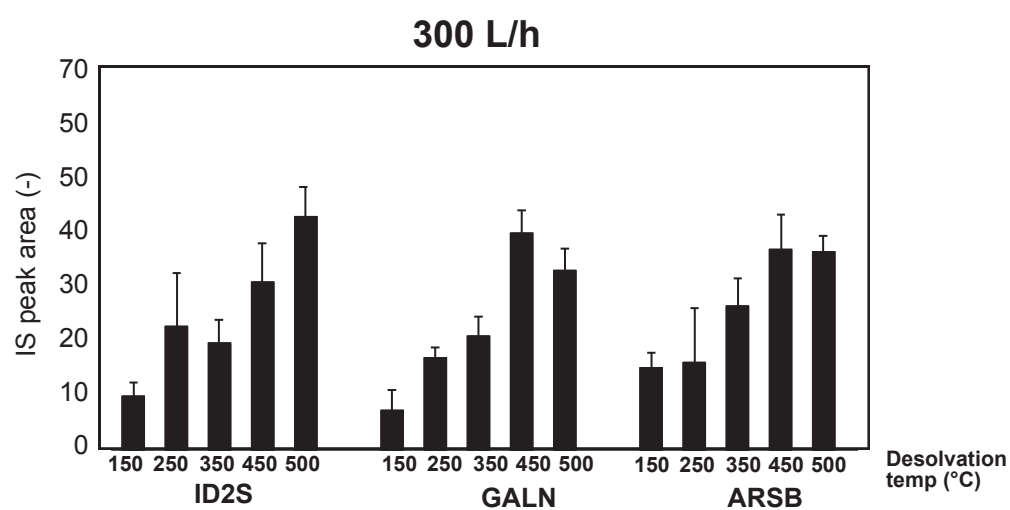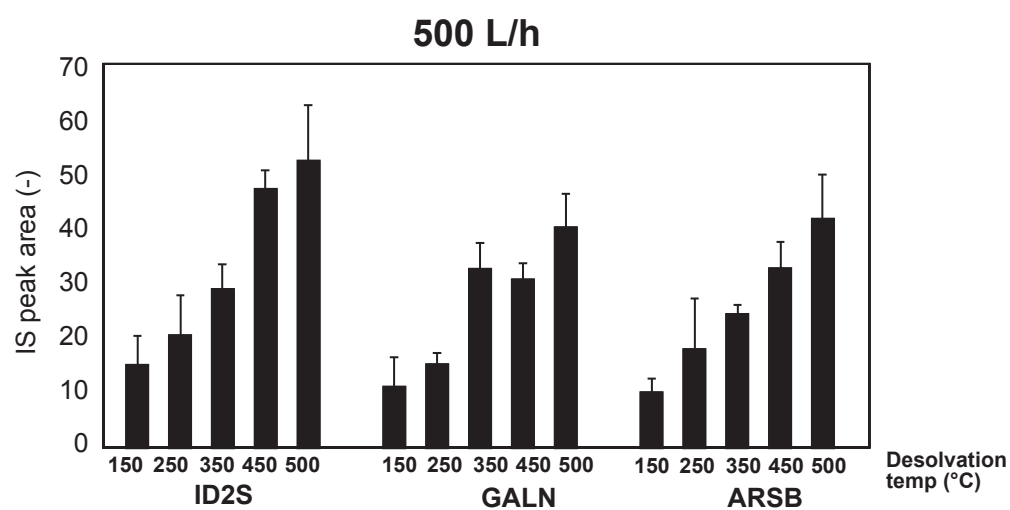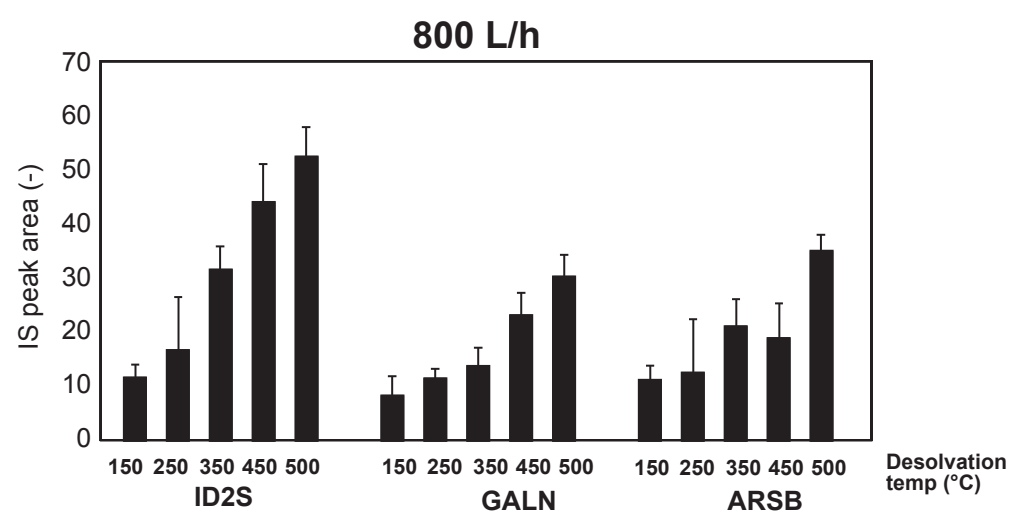

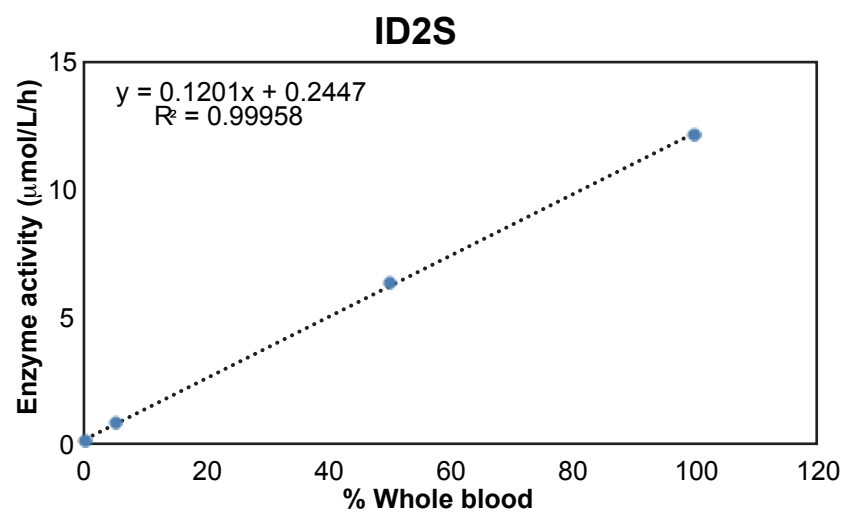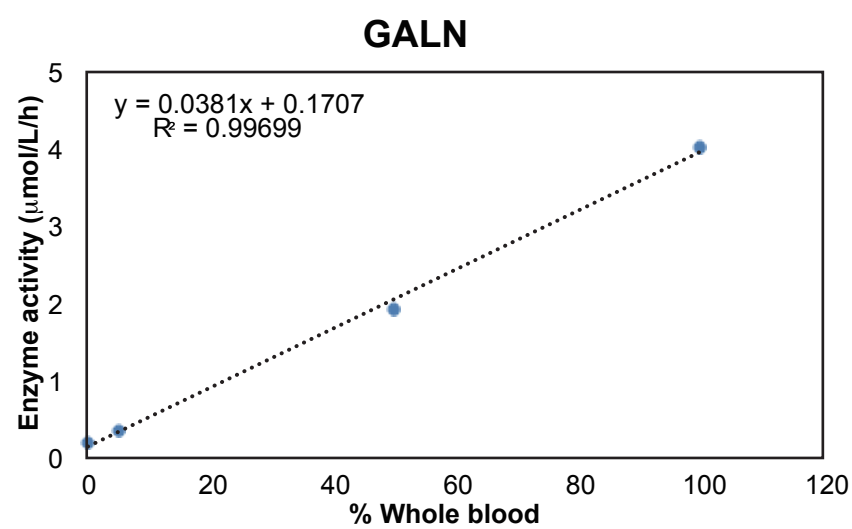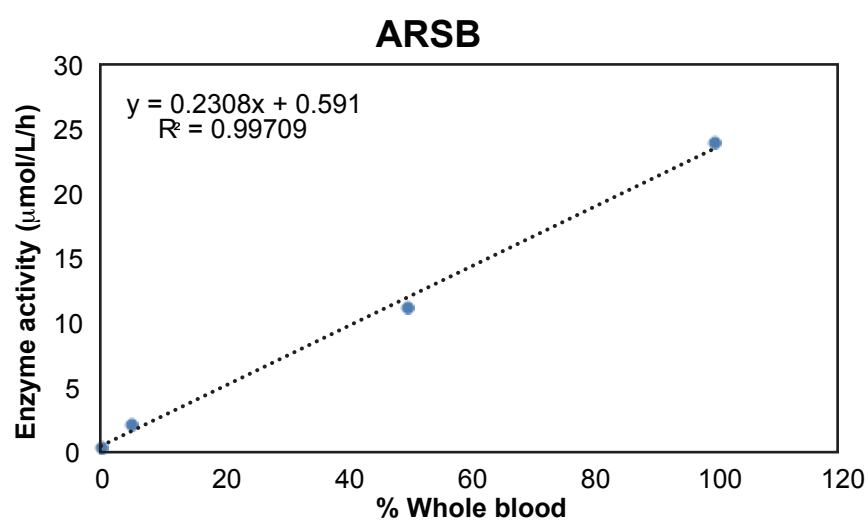

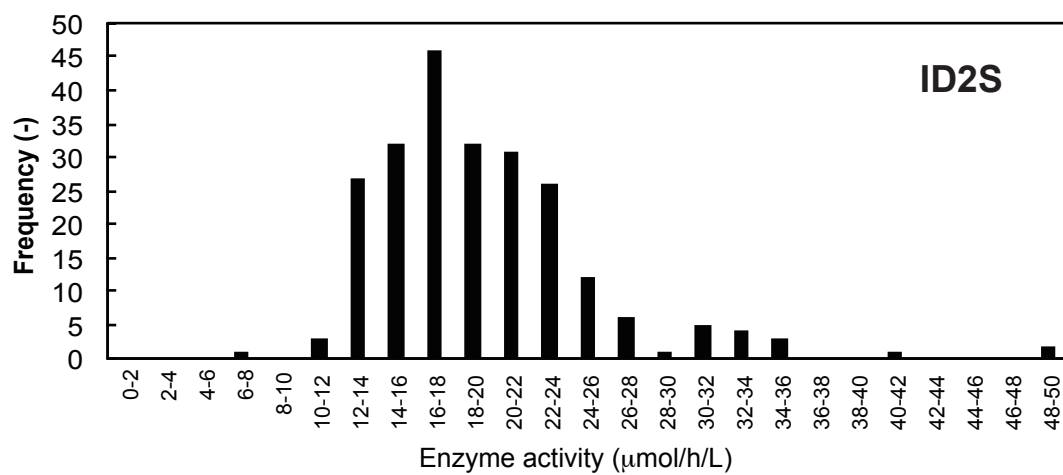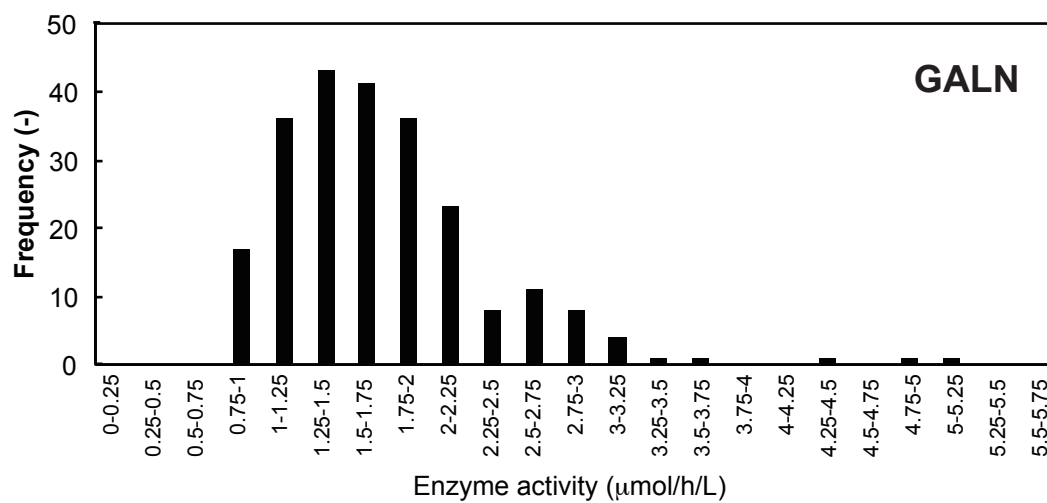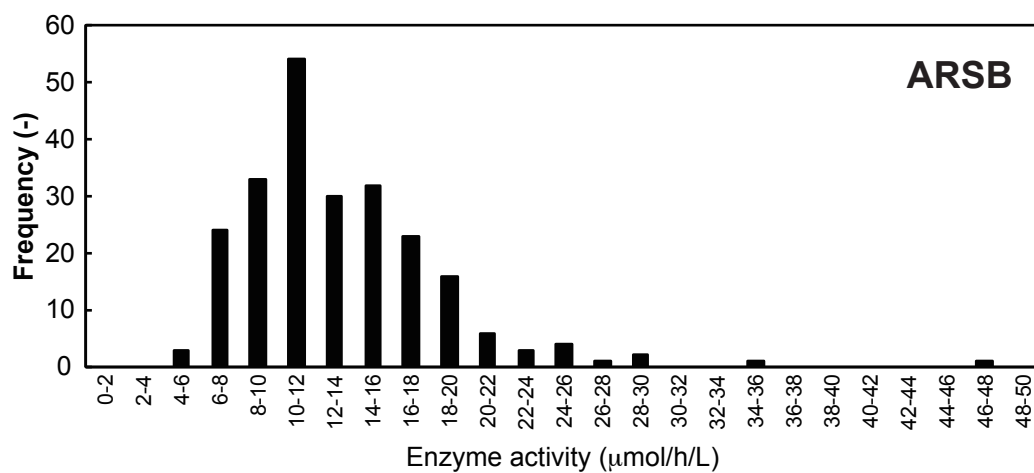

Supplemental Fig. 6  
Mashima R et al.

Supplement: Supplementary file 2 — Supplementary figures [file mmc2.pdf]
